# Elevated VCAM-1, MCP-1 and ADMA serum levels related to pulmonary fibrosis of interstitial lung disease associated with rheumatoid arthritis

**DOI:** 10.3389/fmolb.2022.1056121

**Published:** 2022-12-19

**Authors:** Verónica Pulito-Cueto, Sara Remuzgo-Martínez, Fernanda Genre, Belén Atienza-Mateo, Víctor M. Mora-Cuesta, David Iturbe-Fernández, Leticia Lera-Gómez, María Sebastián Mora-Gil, Diana Prieto-Peña, Virginia Portilla, Ricardo Blanco, Alfonso Corrales, J. Gonzalo Ocejo-Vinyals, Oreste Gualillo, Iván Ferraz-Amaro, José M. Cifrián, Raquel López-Mejías, Miguel A. González-Gay

**Affiliations:** ^1^ Research Group on Genetic Epidemiology and Atherosclerosis in Systemic Diseases and in Metabolic Bone Diseases of the Musculoskeletal System, IDIVAL, Santander, Cantabria, Spain; ^2^ Department of Rheumatology, Hospital Universitario Marqués de Valdecilla, Santander, Cantabria, Spain; ^3^ Department of Pneumology, Hospital Universitario Marqués de Valdecilla, Santander, Cantabria, Spain; ^4^ Department of Immunology, Hospital Universitario Marqués de Valdecilla, Santander, Cantabria, Spain; ^5^ SERGAS (Servizo Galego de Saude) and IDIS (Instituto de Investigación Sanitaria de Santiago), NEIRID Lab. (Neuroendocrine Interactions in rheumatology and inflammatory diseases), Research laboratory 9, Santiago University Clinical Hospital, Santiago de Compostela, Spain; ^6^ Department of Rheumatology, Hospital Universitario de Canarias, Santa Cruz de Tenerife, Spain; ^7^ School of Medicine, Universidad de Cantabria, Santander, Cantabria, Spain; ^8^ Department of medicine and psychiatry, Universidad de Cantabria; Rheumatology division, Hospital Universitario Marqués de Valdecilla; Research group on genetic epidemiology and atherosclerosis in systemic diseases and in metabolic diseases of the musculoskeletal system, IDIVAL, Santander, Cantabria, Spain; ^9^ Cardiovascular Pathophysiology and Genomics Research Unit, School of Physiology, Faculty of Health Sciences, University of the Witwatersrand, Johannesburg, South Africa

**Keywords:** VCAM-1, MCP-1, ADMA, interstitial lung disease, rheumatoid arthritis, biomarkers, pulmonary fibrosis

## Abstract

**Introduction:** Early diagnosis of interstitial lung disease (ILD) associated with rheumatoid arthritis (RA) constitutes a challenge for the clinicians. Pulmonary vasculopathy is relevant in the development of interstitial lung disease. Accordingly, we aimed to explore the role of vascular cell adhesion molecule-1 (VCAM-1), monocyte chemoattractant protein-1 (MCP-1) and asymmetric dimethylarginine (ADMA), key molecules in the vasculopathy, as potential biomarkers of pulmonary fibrosis in RA-ILD^+^.

**Methods:** We included 21 RA-ILD^+^ patients and two comparative groups: 25 RA-ILD^-^ patients and 21 idiopathic pulmonary fibrosis (IPF) patients. Serum levels of the molecules were determined by ELISA, and mRNA expression was quantified by qPCR.

**Results:** VCAM-1, MCP-1 and ADMA serum levels were increased in RA-ILD^+^ patients in relation to RA-ILD^-^ and IPF patients. Additionally, RA-ILD^+^ patients exhibited increased *CCL2* (gene encoding MCP-1) and decreased *PRMT1* (gene related to ADMA synthesis) mRNA expression in relation to RA-ILD^-^ patients. A lower expression of *VCAM1*, *CCL2*, and *PRMT1* was observed in RA-ILD^+^ patients when compared with those with IPF. Furthermore, MCP-1 serum levels and *PRMT1* mRNA expression were positively correlated with RA duration, and ADMA serum levels were positively associated with C-reactive protein in RA-ILD^+^ patients.

**Conclusion:** Our study suggests that VCAM-1, MCP-1 and ADMA could be considered as useful biomarkers to identify ILD in RA patients, as well as to discriminate RA-ILD^+^ from IPF, contributing to the early diagnosis of RA-ILD^+^.

## 1 Introduction

Endothelial dysfunction is characterized by an imbalance in the bioavailability of active substances of endothelial origin, being critical in the development of several autoimmune and inflammatory diseases such as rheumatoid arthritis (RA) (Sattar et al., 2003; González-Gay et al., 2008; Murdaca et al., 2012; Steyers and Miller, 2014; Yang et al., 2016). In this sense, key factors in the vasculopathy and inflammatory response include vascular cell adhesion molecule-1 (VCAM-1), one of the main responsibles of the firm adhesion of leukocytes to the endothelium (Dessein et al., 2005; Navarro-Hernández et al., 2009; Murdaca et al., 2012; Steyers and Miller, 2014; Wang et al., 2015; Kong et al., 2018), and the monocyte chemoattractant protein-1 (MCP-1), a chemokine that regulates the endothelial transmigration of monocyte (Hayashida et al., 2001; Rantapää-Dahlqvist et al., 2007; Deshmane et al., 2009; Dessein et al., 2013; Yang et al., 2016). Besides, asymmetric dimethylarginine (ADMA) is also involved in these disorders, regulating the vascular tone and inhibiting the nitric oxide synthesis (Dimitroulas et al., 2012; Murdaca et al., 2012; Steyers and Miller, 2014; Dimitroulas and Kitas, 2019; Zhao et al., 2019).

Interestingly, pulmonary endothelium dysfunction has also been described as a key stage for the development of lung damage and the subsequent onset and progression of interstitial lung disease (ILD). ILD represents one of the main causes of mortality in patients with RA, being the early detection of pulmonary involvement crucial to avoid an irreversible lung damage in these patients (Gómez-Carrera and Bonilla-Hernan, 2013; Bacha et al., 2017; Hyldgaard et al., 2017; Zamora-legoff et al., 2017; Bendstrup et al., 2019; Manfredi et al., 2021). However, the early diagnosis of RA-ILD^+^ constitutes a challenge for the clinicians because of the absence of symptoms in early or mild disease in some patients as well as the similarity of clinical, pathological, and epidemiological features with idiopathic pulmonary fibrosis (IPF), the most severe ILD (Paulin et al., 2015; Bendstrup et al., 2019; Atienza-Mateo et al., 2020; Manfredi et al., 2021).

In this context, we recently described that endothelial progenitor cells and angiogenic T cells, key cells in the vascular endothelium repair, are involved in the pathogenesis of ILD linked to autoimmune diseases (Pulito-Cueto et al., 2020; Pulito-Cueto et al., 2021; Pulito-Cueto et al., 2022). Hence, it is conceivable to think that other endothelial dysfunction-related markers could also contribute to the early identification of ILD in patients with RA. Nevertheless, this issue is far from being fully elucidated yet.

Accordingly, the aim of this study was to explore the functional role of VCAM-1, MCP-1 and ADMA, assessing both protein and mRNA, as potential biomarkers of pulmonary fibrosis in RA-ILD^+^.

## 2 Materials and methods

### 2.1 Study population

Peripheral venous blood was collected from 21 patients with RA-ILD^+^ and from patients of two comparative groups: 25 patients with RA-ILD^-^ and 21 patients with IPF. All patients were recruited from the Pneumology and Rheumatology departments of Hospital Universitario Marqués de Valdecilla (Santander, Spain).

Patients with RA fulfilled the 2010 American College of Rheumatology criteria for RA (Aletaha et al., 2010). Pulmonary involvement was assessed in all the patients by high-resolution computed tomography (HRCT) images of the chest and pulmonary function tests (PFTs). RA-ILD^-^ patients lacked lung involvement, whereas those with RA-ILD^+^ fulfilled the American Thoracic and European Respiratory Society’s (ATS/ERS) criteria for ILD (Travis et al., 2013). IPF patients fulfilled the ATS/ERS criteria (Travis et al., 2013)*.* HRCT patterns of ILD patients were stratified according to the criteria for usual interstitial pneumonia (UIP) pattern of the Fleischner Society (Lynch et al., 2018).

All procedures involving humans and human blood samples were carried out in accordance with the approved guidelines and regulations, according to the Declaration of Helsinki. All experimental protocols were approved by the Ethics Committee of clinical research of Cantabria, Spain (2016.092). All subjects gave written informed consent to participate in this study before their inclusion.

### 2.2 Serum levels determination

VCAM-1, MCP-1 and ADMA serum levels were determined by a commercial Enzyme-Linked ImmunoSorbent Assay kit (VCAM-1: BMS232, Invitrogen, Vienna, Austria; MCP-1: BMS281, Invitrogen, Vienna, Austria; ADMA: K7860, Immundiagnostik AG, Bensheim, Germany), in accordance with the manufacturer’s instructions. All the samples were analyzed in duplicate and quantified relative to a standard curve, using 5-parameter logistic regression for VCAM-1 and MCP-1 and 4-parameter logistic regression for ADMA through MyAssays^®^ online software as recommended by the manufacturer.

### 2.3 mRNA expression studies

Total RNA was isolated from peripheral blood using the NucleoSpin RNA Blood Kit (Macherey-Nagel, Germany), according to the manufacturer’s instructions. RNA was reverse transcribed into complementary DNA (cDNA) using iScript™ Advanced cDNA Synthesis Kit for reverse transcription-quantitative real-time polymerase chain reaction (qPCR) (Bio-Rad,United States). cDNA was amplified by qPCR in the thermocycler QuantStudio™ 7 Flex Real-Time PCR System (Applied Biosystems, Foster City, CA, United States) using SsoAdvanced™ universal SYBR^®^ Green Supermix (Bio-Rad, United States). All samples were assayed in duplicate and experimental control assays were included. The relative VCAM1, *CCL2* (gene enconding MCP-1) and *PRMT1* (gene enconding the enzyme that catalyzes the ADMA synthesis reaction) mRNA expression was analyzed by the comparative Ct method using GAPDH as housekeeping gene.

### 2.4 Statistical analyses

In the first place, outliers were identified using the formula mean ±2 × standard deviation (SD) and excluded from the analyses. Data were expressed as mean ± SD for continuous variables, and number of individuals (n) and percentage (%) for categorical variables.

Differences related to demographic and clinical features between RA-ILD^+^ and RA-ILD^-^ patients as well as between RA-ILD^+^ and IPF patients were analysed. In particular, sex, smoking ever and antibody status were assessed by chi-squared test, whereas continuous variables were evaluated by Student’s *t*-test.

Comparisons of protein levels or mRNA expression between two study groups were performed through linear regression adjusting for the following potential confounding factors: sex, age at the time of the study, and smoking history. Relationship of protein levels or mRNA expression with continuous and categorical variables was carried out *via* estimation of the Pearson’s partial correlation coefficient (r) and linear regression, respectively, adjusting for the potential confounding factors above mentioned.

The usefulness of serum VCAM-1, MCP-1, and ADMA as diagnostic biomarkers for RA-ILD^+^ was assessed by receiver operating characteristic (ROC) analysis. The area under the curve (AUC) with a 95% confidence interval (CI) was calculated. The optimal cutoff values for discriminating RA-ILD^+^ from RA-ILD^-^ and IPF were calculated by the Youden index, with the maximum value obtained corresponding to the optimal cutoff point.

Statistically significant differences were considered as *p* ≤ 0.05. Statistical analysis was performed using STATA statistical software 12/SE (Stata Corp., College Station, TX, United States).

## 3 Results

### 3.1 Differences in the demographic and clinical features between the study groups

Demographic and clinical features including sex, age, smoking history, PFTs and HRCT patterns were collected from all the patients (Table 1).

**TABLE 1:** Demographic and clinical characteristics of all the patients included in the study.

	RA-ILD^+^ *n* = 21	RA-ILD^−^ *n* = 25	IPF *n* = 21	*p* RA-ILD^+^ vs*.* RA-ILD^−^/RA-ILD^+^ vs*.* IPF
Sex (women), n (%)	9 (45.9)	15 (60.0)	7 (33.3)	0.84/0.53
Age at study (years), mean ± SD	66.5 ± 10.1	60.1 ± 11.8	69.2 ± 10.0	**0.05**/0.38
Smoking ever, n (%)	13 (65.0)	13 (52.0)	16 (76.2)	0.50/0.32
RA duration (years), mean ± SD	9.2 ± 10.2	4.1 ± 7.4	—	0.06/—
CRP (mg/dl), mean ± SD	1.1 ± 1.1	0.5 ± 0.5	—	**0.04**/—
ESR (mm/1st hour), mean ± SD	22.8 ± 27.2	14.4 ± 12.4	—	0.24/—
Disease activity			—	
DAS28-CRP, mean ± SD	3.4 ± 1.4	2.2 ± 1.1	—	**<0.01**/—
DAS28-ESR, mean ± SD	3.4 ± 1.8	2.6 ± 1.6	—	0.12/—
Antibody status			—	
RF^+^, n (%)	17 (81.0)	11 (44.0)	—	**0.01**/—
ACPA^+^, n (%)	19 (90.4)	15 (60.0)	—	**0.02**/—
Pulmonary function tests				
FVC (% predicted), mean ± SD	95.2 ± 24.1	99.2 ± 16.0	84.9 ± 14.7	0.58/0.10
FEV1 (% predicted), mean ± SD	92.2 ± 21.0	94.9 ± 22.0	87.3 ± 19.6	0.67/0.43
FEV1/FVC (% predicted), mean ± SD	77.8 ± 9.1	93.6 ± 12.3	79.7 ± 7.8	**<0.01**/0.47
DLCO (% predicted), mean ± SD	43.3 ± 15.9	79.9 ± 20.0	43.6 ± 18.4	**<0.01**/0.97
HRCT				
Pulmonary involvement	21 (100.0)	0 (0.0)	21 (100.0)	—
UIP pattern, n (%)	11 (52.4)	—	21 (100.0)	—
Probable UIP pattern, n (%)	2 (9.5)	—	0 (0.0)	—
NSIP pattern, n (%)	7 (33.3)	—	0 (0.0)	—
Non-NSIP pattern, n (%)	1 (4.8)	—	0 (0.0)	—

RA, rheumatoid arthritis; ILD, interstitial lung disease; IPF, idiopathic pulmonary fibrosis; SD, standard deviation; CRP, C-reactive protein; ESR, erythrocyte sedimentation rate; DAS, disease activity score; RF, rheumatoid factor; ACPA, anti-cyclic citrullinated peptide antibodies; FVC, forced vital capacity; FEV1, forced expiratory volume at first second; DLCO, diffusing capacity of the lung for carbon monoxide; HRCT, high resolution computed tomography; UIP, usual interstitial pneumonia; NSIP, non-specific interstitial pneumonia. Significant results are highlighted in bold.

Statistically significant differences were found between patients with RA-ILD^+^ and RA-ILD^-^ regarding the following demographic and clinical features: age, C-reactive protein (CRP) levels, disease activity score 28-CRP, rheumatoid factor and anti-cyclic citrullinated peptide antibodies status (Table 1). Likewise, we also observed differences in forced expiratory volume at first second/forced vital capacity and diffusing capacity of the lung for carbon monoxide (Table 1). However, no differences were disclosed between RA-ILD^+^ and IPF patients (Table 1).

### 3.2 VCAM-1, MCP-1 and ADMA associated with RA-ILD^+^

**FIGURE 1:**
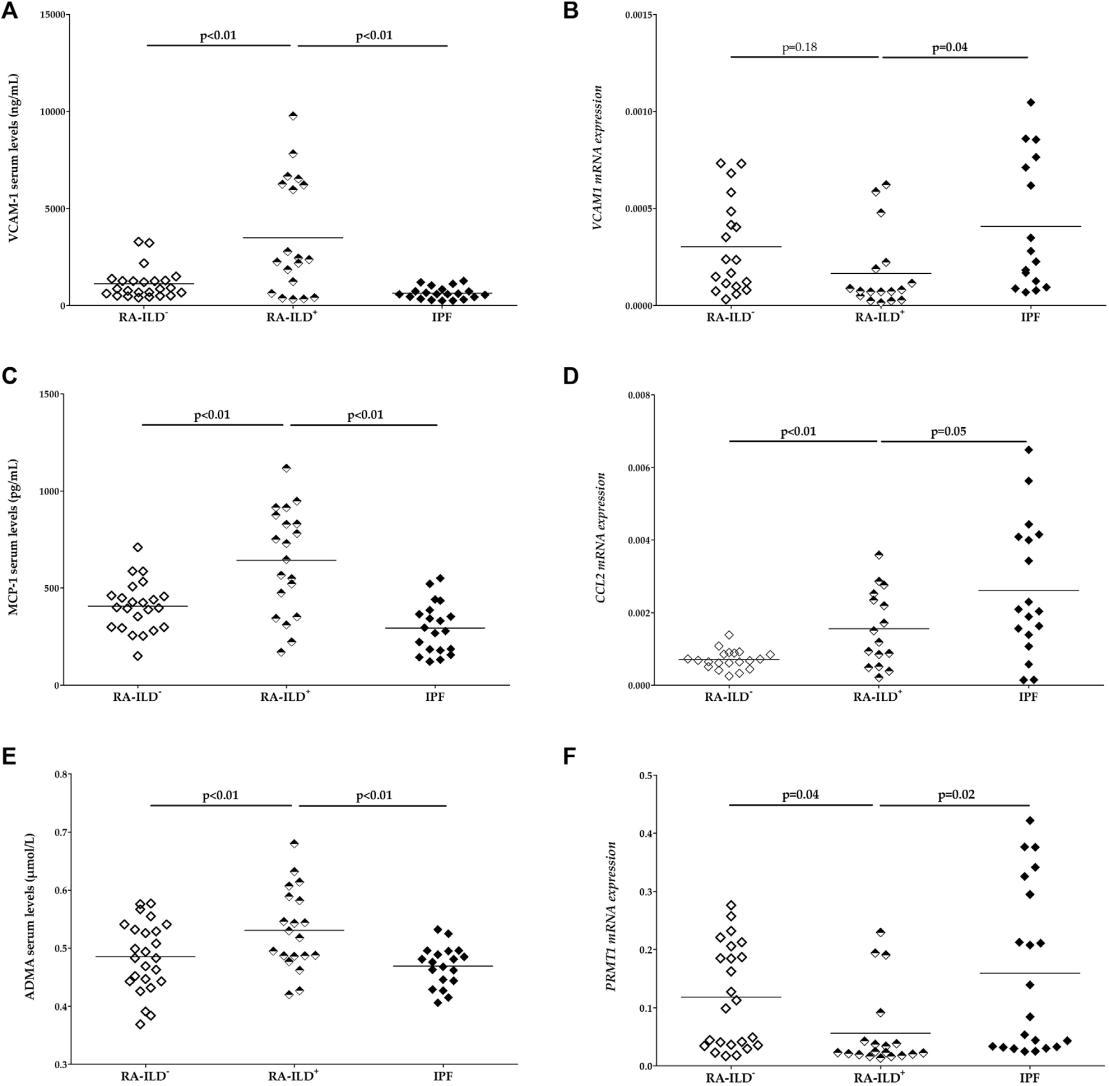
Differences in serum levels of VCAM-1 **(A)**, MCP-1 **(C)** and ADMA **(E)** as well as mRNA expression of *VCAM1* **(B)**, *CCL2* **(D)** and *PRMT1* **(F)** between RA-ILD^+^ patients and the comparative groups. RA: rheumatoid arthritis; ILD, interstitial lung disease; IPF, idiopathic pulmonary fibrosis; VCAM-1, vascular cell adhesion molecule 1; MCP-1, monocyte chemoattractant protein-1; ADMA, asymmetric dimethylarginine. Significant results are highlighted in bold.

On the one hand, VCAM-1, MCP-1 and ADMA levels were increased in patients with RA-ILD^+^ in relation to RA-ILD^-^ patients (*p* < 0.01 in all cases, Figures 1A,C,E, Supplementary Table S1). Moreover, patients with RA-ILD^+^ exhibited a higher *CCL2* mRNA expression than RA-ILD^-^ patients (*p* < 0.01, Figure 1D, Supplementary Table S1). Furthermore, a lower *PRMT1* mRNA expression was found in patients with RA-ILD^+^ in comparison with their negative counterpants (*p* = 0.04, Figure 1F, Supplementary Table S1).

On the other hand, patients with RA-ILD^+^ showed significantly higher serum levels of VCAM-1, MCP-1 and ADMA compared to those with IPF (*p* < 0.01 in all cases, Figures 1A, C, E, Supplementary Table S1). Additionally, a lower expression of *VCAM1*, *CCL2*, and *PRMT1* was observed in patients with RA-ILD^+^ when compared with those with IPF (*p* = 0.04, *p* = 0.05, *p* = 0.02, respectively, Figures 1B, D, F, Supplementary Table S1).

### 3.3 VCAM-1, MCP-1 and ADMA as diagnostic biomarkers for RA-ILD^+^

**FIGURE 2:**
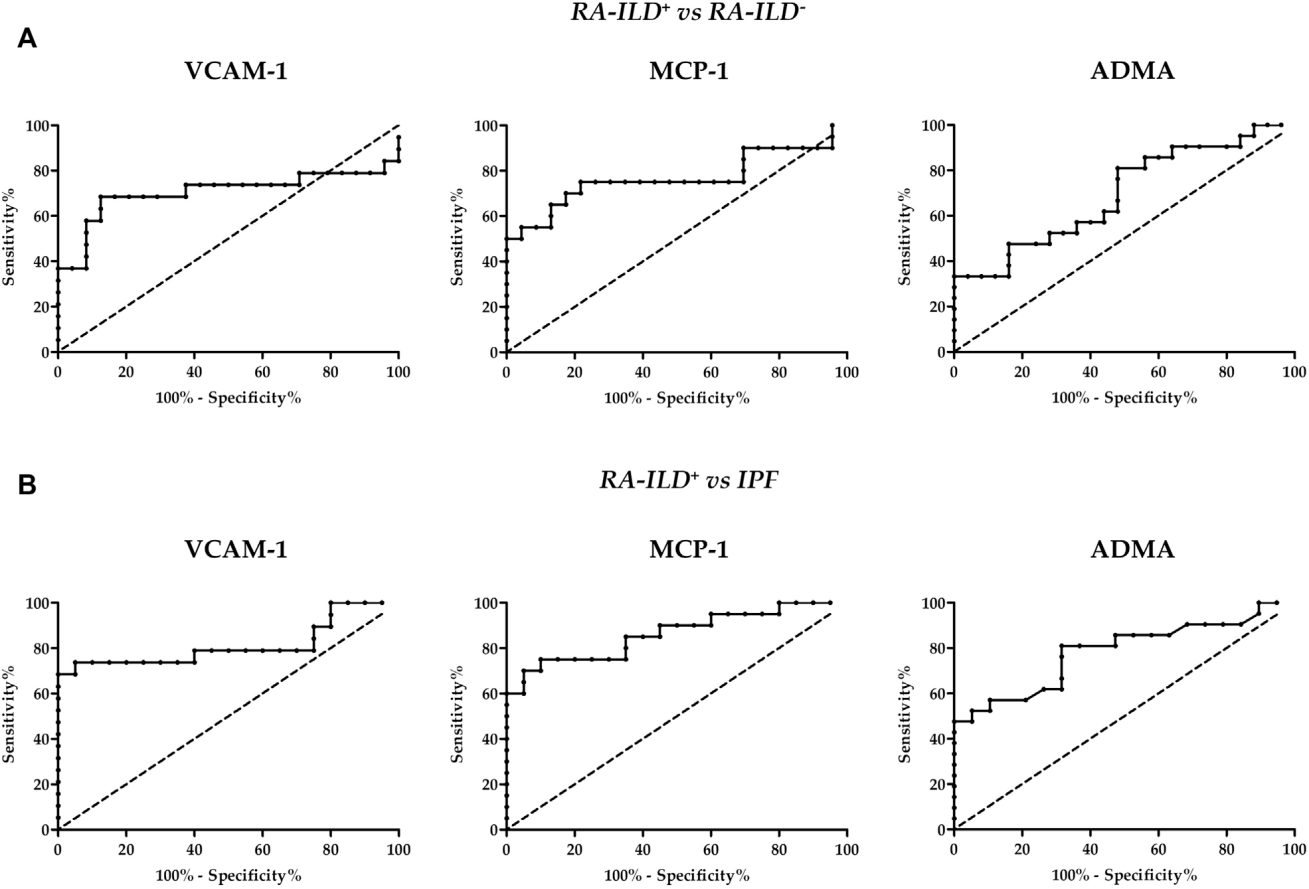
ROC curves of VCAM-1, MCP-1 and ADMA for the screening of RA-ILD^+^ and RA-ILD^−^ **(A)** and RA-ILD^+^ and IPF **(B)**. RA, rheumatoid arthritis; ILD, interstitial lung disease; IPF, idiopathic pulmonary fibrosis; VCAM-1, vascular cell adhesion molecule 1; MCP-1, monocyte chemoattractant protein-1; ADMA, asymmetric dimethylarginine.

To evaluate the ability of serum VCAM-1, MCP-1 and ADMA levels for discriminating patients with RA-ILD^+^ from those with RA-ILD^-^, ROC curves were drawn for each biomarker (Figure 2A). Interestingly, the AUC was 0.704, 0.765 and 0.695, respectively (Table 2). The optimal cutoff value for VCAM-1, MCP-1 and ADMA showing the best sensitivity and specificity was 1680.0 ng/ml, 468.2 pg/ml and 0.5795 μmol/L, respectively (Table 2).

**TABLE 2:** Receiver operating characteristic curves analysis for the discrimination of RA-ILD^+^ from RA-ILD^−^ and IPF.

	AUC (95% CI)	Optimal cutoff value	Sensitivity (%)	Specificity (%)
RA-ILD^+^ vs. RA-ILD^−^				
VCAM-1	0.704 (0.520–0.888)	1680.0 ng/ml	68.4	87.5
MCP-1	0.765 (0.607–0.923)	468.2 pg/ml	75.0	78.3
ADMA	0.695 (0.542–0.849)	0.5795 μmol/L	33.3	100.0
RA-ILD^+^ vs. IPF				
VCAM-1	0.813 (0.663–0.964)	1220.0 ng/ml	73.7	95.0
MCP-1	0.863 (0.746–0.979)	458.4 pg/ml	75.0	90.0
ADMA	0.783 (0.638–0.927)	0.4855 μmol/L	81.0	68.4

RA, rheumatoid arthritis; ILD: interstitial lung disease; IPF, idiopathic pulmonary fibrosis; AUC, area under the curve; CI, confidence interval; VCAM-1, vascular cell adhesion molecule 1; MCP-1, monocyte chemoattractant protein-1; ADMA, asymmetric dimethylarginine.

In addition, ROC curves were used for the differentiation of patients with RA-ILD^+^ from those with IPF (Figure 2B). Of note, the AUC was 0.813 for VCAM-1, 0.863 for MCP-1 and 0.783 for ADMA (Table 2). The optimal cutoff value was 1220.0 ng/ml, 458.4 pg/ml and 0.4855 μmol/L, respectively (Table 2).

### 3.4 Lack of association of VCAM-1, MCP-1 and ADMA with clinical characteristics related to pulmonary involvement of RA-ILD^+^ patients

No relationship between VCAM-1, MCP-1 and ADMA protein levels and PFTs as well as HRCT patterns of patients with RA-ILD^+^ was found (Supplementary Table S2). Likewise, no significant results were obtained regarding *VCAM1*, *CCL2* and *PRMT1* mRNA expression (Supplementary Table S2).

### 3.5 Relationship of VCAM-1, MCP-1 and ADMA with clinical characteristics intrinsic of the rheumatic disease in RA-ILD^+^ and RA-ILD^−^ patients

**FIGURE 3:**
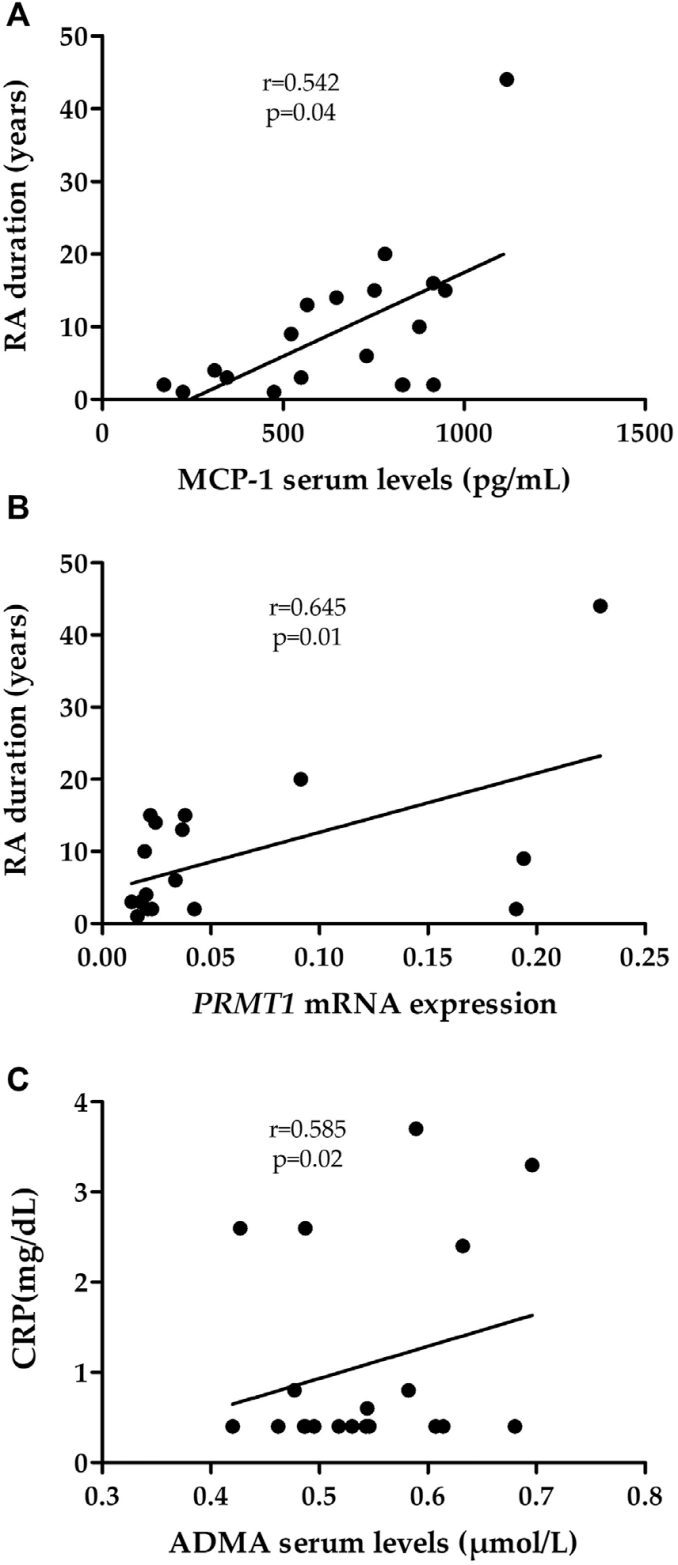
Relationship of MCP-1 **(A)**, *PRMT1* mRNA expression **(B)** and ADMA **(C)** with clinical characteristics of RA-ILD^+^ patients. RA, rheumatoid arthritis; MCP-1, monocyte chemoattractant protein-1; CRP, C-reactive protein; ADMA, asymmetric dimethylarginine.

Regarding RA-ILD^+^ patients, a positive partial correlation was found between MCP-1 levels and RA duration (*r* = 0.542, *p* = 0.037, Figure 3A, Supplementary Table S3). This was also the case when *PRMT1* mRNA expression was assessed (*r* = 0.645, *p* = 0.013, Figure 3B, Supplementary Table S3). In addition, ADMA serum levels were positively partially correlated with CRP (*r* = 0.585, *p* = 0.022, Figure 3C, Supplementary Table S3). No relationship between VCAM-1, MCP-1 and ADMA protein levels and other clinical characteristics of patients with RA-ILD^+^ were found (Supplementary Table S3). Likewise, no other significant results were obtained regarding *VCAM1*, *CCL2* and *PRMT1* mRNA expression (Supplementary Table S3).

With respect to patients with RA-ILD^-^, no association was found between VCAM-1, MCP-1 and ADMA and clinical characteristics intrinsic of RA, both when assessing protein levels and mRNA expression (Supplementary Table S4).

## 4 Discussion

Endothelial dysfunction, an early stage in the vascular damage, is observed in patients with RA (González-Gay et al., 2008) and is also implicated in ILD (Gómez-Carrera and Bonilla-Hernan, 2013; Bacha et al., 2017; Hyldgaard et al., 2017; Zamora-legoff et al., 2017; Bendstrup et al., 2019; Manfredi et al., 2021). RA-ILD^+^ diagnosis is often delayed due to the heterogeneity and unpredictability of ILD, constituting a major concern for the clinicians (Paulin et al., 2015; Bendstrup et al., 2019; Atienza-Mateo et al., 2020; Manfredi et al., 2021).

Given that pulmonary vasculopathy is important for the development of ILD, endothelial dysfunction-related markers could help to the early identification of RA-ILD^+^. Interestingly, this study provides evidence, for the first time, of an association of VCAM-1, MCP-1 and ADMA with pulmonary fibrosis in RA-ILD^+^.

First, we found that VCAM-1 serum levels were increased in patients with RA-ILD^+^ in relation to those with RA-ILD^-^ and IPF. In this context, it has been described that VCAM-1 is induced by both inflammatory cytokines and hypoxia (Peter et al., 1999; Dessein et al., 2005; Navarro-Hernández et al., 2009; Cook-Mills et al., 2011; Murdaca et al., 2012; Wang et al., 2015; Kong et al., 2018; McGroder et al., 2019), which may explain the increase of this protein observed in our RA-ILD^+^ patients. Likewise, its ability to stimulate the fibroblast proliferation may be related to the fibrotic properties of this disease (Agassandian et al., 2015; McGroder et al., 2019). Moreover, our findings revealed a decrease of *VCAM1* expression in RA-ILD^+^ patients with respect to the comparative groups, suggesting the involvement of post-transcriptional regulatory mechanisms (Greenbaum et al., 2003; Stevens and Brown, 2013; Li et al., 2020).

In the second place, we also disclosed a relationship of MCP-1 with lung involvement in RA since patients with RA-ILD^+^ presented higher levels of MCP-1 than those with RA-ILD^−^ and IPF. Of note, we noticed that information on the role of MCP-1 in RA-ILD^+^ was limited to a single study in Asian population that also showed the same differences between RA-ILD^+^ and RA-ILD^−^ patients (Ling et al., 2010). In this regard, a profibrotic role of MCP-1 has been previously described in other autoimmune diseases such as systemic sclerosis (Hasegawa et al., 1999; Schmidt et al., 2009; Yalçinkaya et al., 2016). The higher levels of MCP-1 in RA-ILD^+^ may also be caused by the chronic systemic exposure to proinflammatory cytokines characteristic of RA (Hayashida et al., 2001; Rantapää-Dahlqvist et al., 2007; Deshmane et al., 2009; Lin et al., 2014). It is noteworthy that an increase of *CCL2* expression was observed in our patients with RA-ILD^+^ compared to those with RA-ILD^−^, whereas IPF patients showed the highest *CCL2* expression. These results could indicate that *CCL2* expression increases in response to ILD severity, supporting its known profibrotic role (Hasegawa et al., 1999; Schmidt et al., 2009; Ling et al., 2010). Moreover, we found a positive correlation between MCP-1 serum levels and RA duration in RA-ILD^+^ patients, which is in line with its key endothelial function reported in RA (Hayashida et al., 2001; Rantapää-Dahlqvist et al., 2007; Deshmane et al., 2009; Lin et al., 2014).

Finally, RA-ILD^+^ patients showed an increase of ADMA serum levels compared to both RA-ILD^−^ and IPF patients, pointing out ADMA as a biomarker of RA-ILD^+^. The highest ADMA levels in RA-ILD^+^ patients make sense considering that ADMA has been previously described as a contributor of lung fibrosis and that its degradation may be inhibited by hypoxia and inflammatory cytokines, common processes of this disease (Ito et al., 1999; Millatt et al., 2003; Sattar et al., 2003; Sandoo et al., 2012; Janssen et al., 2013). Indeed, a positive correlation of ADMA and CRP was found in our cohort of RA-ILD^+^ patients. It should also be mentioned that RA-ILD^+^ patients presented the lowest *PRMT1* expression, probably as a result of post-transcriptional regulatory mechanisms (Greenbaum et al., 2003; Stevens and Brown, 2013; Li et al., 2020). Furthermore, *PRMT1* expression was positively correlated with RA duration in RA-ILD^+^ patients, confirming its previously described relationship with RA (Sandoo et al., 2012; Dimitroulas and Kitas, 2019; Zhao et al., 2019).

Considering all these findings, we can hypothesize that an additive effect combining the vascular damage caused by chronic inflammation and other features of RA as well as that associated with the fibrotic processes typical of ILD is related to increased serum concentrations of VCAM-1, MCP-1 and ADMA in RA-ILD^+^. In addition, serum levels of VCAM-1, MCP-1 and ADMA higher than 1680.0 ng/ml, 468.2 pg/ml and 0.5795 μmol/L may differentiate RA-ILD^+^ from RA-ILD^-^. Likewise, serum levels higher than 1220.0 ng/ml, 458.4 pg/ml and 0.4855 μmol/L, respectively, may discriminate RA-ILD^+^ from IPF. These results could be relevant in the clinical practice for the diagnosis of RA-ILD^+^.

In conclusion, our study suggests that VCAM-1, MCP-1 and ADMA can be useful biomarkers to identify the presence of ILD in patients with RA, as well as to discriminate between RA-ILD^+^ and IPF, contributing to the early diagnosis of RA-ILD^+^.

## Data Availability

The original contributions presented in the study are included in the article/Supplementary Material, further inquiries can be directed to the corresponding author.

## Abbreviations

ACPA, anti-cyclic citrullinated peptide antibodies; ADMA, asymmetric dimethylarginine; ATS/ERS, American Thoracic and European Respiratory Society’s; CRP, C-reactive protein; DAS, diasease activity score; DLCO, diffusing capacity of the lung for carbon monoxide; ESR, erythrocyte sedimentation rate; FEV1, forced expiratory volume at first second; FVC, forced vital capacity; HRCT, high resolution computed tomography; ILD, interstitial lung disease; IPF, idiopathic pulmonary fibrosis; MCP-1, monocyte chemoattractant protein-1; NSIP, non-specific interstitial pneumonia; PFTs, pulmonary function tests; RA, rheumatoid arthritis; RF, rheumatoid factor; SD, standard deviation; UIP, usual interstitial pneumonia; VCAM-1, vascular cell adhesion molecule-1.
