# Supplementary material for: Elevated VCAM-1, MCP-1 and ADMA serum levels related to pulmonary fibrosis of interstitial lung disease associated with rheumatoid arthritis
Source: Front Mol Biosci. 2022 Dec 19;9:1056121. doi: 10.3389/fmolb.2022.1056121 (PMC9806218; doi:10.3389/fmolb.2022.1056121)
Supplement: Supplementary file 1 [file Table1.DOCX]

Supplementary Material

| Supplementary Table S1. VCAM-1, MCP-1 and ADMA serum levels as well as *VCAM1, CCL2* and *PRMT1* mRNA expression in all study groups included in this study. | | | |
| --- | --- | --- | --- |
|  | **RA-ILD^+^** | **RA-ILD^-^** | **IPF** |
| *Protein levels* |  |  |  |
| VCAM-1 (ng/mL), mean ± SD | 3499,0 ± 2978,0 | 1118,0 ± 787,5 | 646,2 ± 313,3 |
| MCP-1 (pg/mL), mean ± SD | 642,8 ± 268,6 | 406,9 ± 127,8 | 295,0 ± 130,1 |
| ADMA (µmol/L), mean ± SD | 0,5307 ± 0,0694 | 0,4852 ± 0,0602 | 0,4693 ± 0,0347 |
|  |  |  |  |
| *mRNA Expression* |  |  |  |
| *VCAM1,* mean ± SD | 0,0002 ± 0,0002 | 0,0003 ± 0,0002 | 0,0004 ± 0,0003 |
| *CCL2,* mean ± SD | 0,0016 ± 0,0010 | 0,0007 ± 0,0002 | 0,0026 ± 0,0018 |
| *PRMT1,* mean ± SD | 0,0565 ± 0,0685 | 0,1180 ± 0,0886 | 0,1591 ± 0,1435 |
| VCAM-1: vascular cell adhesion molecule 1; MCP-1: monocyte chemoattractant protein-1; ADMA: asymmetric dimethylarginine; RA: rheumatoid arthritis; ILD: interstitial lung disease; IPF: idiopathic pulmonary fibrosis; SD: standard deviation. | | | |
