# Supplementary material for: Elevated VCAM-1, MCP-1 and ADMA serum levels related to pulmonary fibrosis of interstitial lung disease associated with rheumatoid arthritis
Source: Front Mol Biosci. 2022 Dec 19;9:1056121. doi: 10.3389/fmolb.2022.1056121 (PMC9806218; doi:10.3389/fmolb.2022.1056121)
Supplement: Supplementary file 2 [file Table2.DOCX]

| Supplementary Table S2. Relationship of VCAM-1, MCP-1 and ADMA serum levels as well as *VCAM1*, *CCL2* and *PRMT1* mRNA expression with PFTs and HRCT pattern of RA-ILD^+^ patients. | | | | | | | | | | | | |  |  |  |
| --- | --- | --- | --- | --- | --- | --- | --- | --- | --- | --- | --- | --- | --- | --- | --- |
|  | **VCAM-1**  **serum levels** | | ***VCAM1***  **mRNA expression** | | **MCP-1**  **serum levels** | | ***CCL2***  **mRNA expression** | | **ADMA**  **serum levels** | | ***PRMT1***  **mRNA expression** | |  |  |  |
| *Variable* | *r* | *p* | *r* | *p* | *r* | *p* | *r* | *p* | *r* | *p* | *r* | *p* |  |  |  |
| FVC (% predicted) | 0.323 | 0.26 | -0.178 | 0.56 | 0.095 | 0.74 | 0.249 | 0.44 | 0.116 | 0.67 | 0.484 | 0.08 |  |  |  |
| FEV1 (% predicted) | 0.166 | 0.57 | -0.266 | 0.38 | 0.060 | 0.83 | 0.219 | 0.50 | 0.048 | 0.86 | 0.463 | 0.10 |  |  |  |
| FEV1/FVC (% predicted) | -0.475 | 0.09 | 0.063 | 0.84 | -0.145 | 0.61 | -0.157 | 0.63 | -0.289 | 0.28 | -0.443 | 0.11 |  |  |  |
| DLCO (% predicted) | 0.614 | 0.08 | 0.477 | 0.19 | 0.426 | 0.25 | 0.076 | 0.90 | 0.343 | 0.37 | 0.017 | 0.97 |  |  |  |
|  |  |  |  | |  |  |  | |  |  |  | |  |  |  |
| *Category* | *Mean ± SD*  *(ng/mL)* | *p* | *Mean ± SD* | *p* | *Mean ± SD*  *(pg/mL)* | *p* | *Mean ± SD* | *P* | *Mean ± SD*  *(µmol/L)* | *p* | *Mean ± SD* | *p* |  |  |  |
| UIP HRCT pattern | 3206.283 ± 2641.529 | 0.37 | 0.00016 ± 0.00020 | 0.97 | 700.483 ± 254.547 | 0.66 | 0.00122 ± 0.00136 | 0.80 | 0.546 ± 0.090 | 0.64 | 0.06015 ± 0.06978 | 0.91 |  |  |  |
| NSIP HRCT pattern | 4614.567 ± 3587.435 |  | 0.00021 ± 0.00026 |  | 605.417 ± 2477.881 |  | 0.00187 ± 0.00092 |  | 0.539 ± 0.079 |  | 0.06147 ± 0.08845 |  |  |  |  |
| VCAM-1: vascular cell adhesion molecule 1; MCP-1: monocyte chemoattractant protein-1; ADMA: asymmetric dimethylarginine; PFTs: pulmonary function tests; HRCT: high resolution computed tomography; RA: rheumatoid arthritis; ILD: interstitial lung disease; FVC: forced vital capacity; FEV1: forced expiratory volume at first second; DLCO: diffusing capacity of the lung for carbon monoxide; UIP: usual interstitial pneumonia; NSIP: non-specific interstitial pneumonia. | | | | | | | | | | | | |  |  |  |

***Supplementary Material***

| Supplementary Table S3. Relationship of VCAM-1, MCP-1 and ADMA serum levels as well as *VCAM1*, *CCL2* and *PRMT1* mRNA expression with clinical characteristics related to rheumatic involvement of RA-ILD^+^ patients. | | | | | | | | | | | | |  |  |  |
| --- | --- | --- | --- | --- | --- | --- | --- | --- | --- | --- | --- | --- | --- | --- | --- |
|  | **VCAM-1**  **serum levels** | | ***VCAM1***  **mRNA Expression** | | **MCP-1**  **Serum levels** | | ***CCL2***  **mRNA Expression** | | **ADMA**  **serum levels** | | ***PRMT1***  **mRNA Expression** | |  |  |  |
| *Variable* | *r* | *p* | *r* | *p* | *r* | *p* | *r* | *p* | *r* | *p* | *r* | *p* |  |  |  |
| Duration of RA (years) | 0.157 | 0.59 | -0.291 | 0.34 | **0.542** | **0.04** | 0.446 | 0.15 | 0.325 | 0.22 | **0.645** | **0.01** |  |  |  |
| CRP (mg/dL) | 0.198 | 0.52 | -0.022 | 0.95 | 0.233 | 0.42 | 0.164 | 0.63 | **0.585** | **0.02** | 0.406 | 0.17 |  |  |  |
| ESR (mm/1^st^ hour) | 0.132 | 0.67 | 0.119 | 0.71 | 0.237 | 0.42 | -0.119 | 0.73 | 0.190 | 0.50 | 0.043 | 0.89 |  |  |  |
| DAS28-CRP | 0.423 | 0.13 | 0.108 | 0.72 | 0.270 | 0.33 | 0.030 | 0.92 | 0.443 | 0.09 | 0.229 | 0.43 |  |  |  |
| DAS28-ESR | 0.349 | 0.22 | 0.119 | 0.70 | 0.228 | 0.41 | -0.055 | 0.87 | 0.389 | 0.14 | 0.244 | 0.40 |  |  |  |
|  |  |  |  | |  |  |  | |  |  |  | |  |  |  |
| *Category* | *Mean ± SD*  *(ng/mL)* | *p* | *Mean ± SD* | *p* | *Mean ± SD*  *(pg/mL)* | *p* | *Mean ± SD* | *P* | *Mean ± SD*  *(µmol/L)* | *p* | *Mean ± SD* | *p* |  |  |  |
| RF^-^ | 1628.467 ± 1131.011 | 0.15 | 0.00015 ± 0.00011 | 0.42 | 583.600 ± 360.171 | 0.47 | 0.00085 ± 0.00048 | 0.65 | 0.489 ± 0.028 | 0.27 | 0.02729 ± 0.00948 | 0.15 |  |  |  |
| RF^+^ | 4024.387 ± 3132.251 |  | 0.00019 ± 0.00021 |  | 672.500 ± 257.898 |  | 0.00157 ± 0.00106 |  | 0.550 ± 0.080 |  | 0.06490 ± 0.07529 |  |  |  |  |
| ACPA^-^ | 6256 .000 | 0.54 | 0.00048 | 0.09 | 831.000 | 0.88 | - | - | 0.544 | 0.84 | 0.04247 | 0.79 |  |  |  |
| ACPA^+^ | 3470 ± 3030.412 |  | 0.00015 ± 0.00019 |  | 648.878 ± 270.692 |  | 0.00148 ± 0.00102 |  | 0.541 ± 0.080 |  | 0.05959 ± 0.07185 |  |  |  |  |
| VCAM-1: vascular cell adhesion molecule 1; MCP-1: monocyte chemoattractant protein-1; ADMA: asymmetric dimethylarginine; RA: rheumatoid arthritis; ILD: interstitial lung disease; CRP: C-reactive protein; ESR: erythrocyte sedimentation rate; DAS: disease activity score; RF: rheumatoid factor; ACPA: anti-cyclic citrullinated peptide antibodies. Significant results are highlighted in bold. | | | | | | | | | | | | |  |  |  |

| Supplementary Table S4. Relationship of VCAM-1, MCP-1 and ADMA serum levels as well as *VCAM1*, *CCL2* and *PRMT1* mRNA expression with clinical characteristics related to rheumatic involvement of RA-ILD^-^ patients. | | | | | | | | | | | | |  |  |  |
| --- | --- | --- | --- | --- | --- | --- | --- | --- | --- | --- | --- | --- | --- | --- | --- |
|  | **VCAM-1**  **serum levels** | | ***VCAM1***  **mRNA Expression** | | **MCP-1**  **Serum levels** | | ***CCL2***  **mRNA Expression** | | **ADMA**  **serum levels** | | ***PRMT1***  **mRNA Expression** | |  |  |  |
| *Variable* | *r* | *p* | *r* | *p* | *r* | *p* | *r* | *p* | *r* | *p* | *r* | *p* |  |  |  |
| Duration of RA (years) | -0.169 | 0.47 | 0.431 | 0.10 | 0.041 | 0.86 | -0.055 | 0.83 | -0.318 | 0.15 | 0.328 | 0.15 |  |  |  |
| CRP (mg/dL) | 0.332 | 0.14 | 0.247 | 0.36 | 0.082 | 0.73 | -0.095 | 0.72 | 0.091 | 0.69 | 0.145 | 0.53 |  |  |  |
| ESR (mm/1^st^ hour) | 0.167 | 0.47 | 0.015 | 0.96 | -0.097 | 0.68 | 0.275 | 0.30 | -0.0007 | 1.00 | -0.026 | 0.91 |  |  |  |
| DAS28-CRP | 0.024 | 0.92 | 0.245 | 0.36 | 0.371 | 0.11 | 0.103 | 0.69 | 0.073 | 0.75 | 0.145 | 0.53 |  |  |  |
| DAS28-ESR | -0.015 | 0.95 | 0.155 | 0.57 | 0.189 | 0.42 | 0.297 | 0.25 | 0.031 | 0.89 | 0.131 | 0.57 |  |  |  |
|  |  |  |  | |  |  |  | |  |  |  | |  |  |  |
| *Category* | *Mean ± SD*  *(ng/mL)* | *p* | *Mean ± SD* | *p* | *Mean ± SD*  *(pg/mL)* | *p* | *Mean ± SD* | *P* | *Mean ± SD*  *(µmol/L)* | *p* | *Mean ± SD* | *p* |  |  |  |
| RF^-^ | 1284.093 ± 959.549 | 0.11 | 0.00346 ± 0.00026 | 0.31 | 390.808 ± 142.871 | 0.37 | 0.00075 ± 0.00028 | 0.34 | 0.497 ± 0.059 | 0.22 | 0.120 ± 0.097 | 0.91 |  |  |  |
| RF^+^ | 884.690 ± 388.974 |  | 0.00021 ± 0.00018 |  | 427.780 ± 108.866 |  | 0.00066 ± 0.00025 |  | 0.470 ± 0.061 |  | 0.116 ± 0.082 |  |  |  |  |
| ACPA^-^ | 966.990 ± 559.209 | 0.81 | 0.00035 ± 0.00029 | 0.19 | 434.044 ± 140.013 | 0.41 | 0.00074 ± 0.00036 | 0.87 | 0.498 ± 0.063 | 0.18 | 0.142 ± 0.101 | 0.34 |  |  |  |
| ACPA^+^ | 1225.307 ± 922.293 |  | 0.00026 ± 0.00019 |  | 389.421 ± 121.402 |  | 0.00069 ± 0.00023 |  | 0.477 ± 0.059 |  | 0.104 ± 0.081 |  |  |  |  |
| VCAM-1: vascular cell adhesion molecule 1; MCP-1: monocyte chemoattractant protein-1; ADMA: asymmetric dimethylarginine; RA: rheumatoid arthritis; ILD: interstitial lung disease; CRP: C-reactive protein; ESR: erythrocyte sedimentation rate; DAS: disease activity score; RF: rheumatoid factor; ACPA: anti-cyclic citrullinated peptide antibodies. Significant results are highlighted in bold. | | | | | | | | | | | | |  |  |  |
