# Supplementary material for: Elevated VCAM-1, MCP-1 and ADMA serum levels related to pulmonary fibrosis of interstitial lung disease associated with rheumatoid arthritis
Source: Front Mol Biosci. 2022 Dec 19;9:1056121. doi: 10.3389/fmolb.2022.1056121 (PMC9806218; doi:10.3389/fmolb.2022.1056121)
Supplement: Supplementary file 3 [file Table3.DOCX]

***Supplementary Material***

| Supplementary Table S3. Relationship of VCAM-1, MCP-1 and ADMA serum levels as well as *VCAM1*, *CCL2* and *PRMT1* mRNA expression with clinical characteristics intrinsic of the rheumatic disease in RA-ILD^+^ patients. | | | | | | | | | | | | |  |  |  |
| --- | --- | --- | --- | --- | --- | --- | --- | --- | --- | --- | --- | --- | --- | --- | --- |
|  | **VCAM-1**  **serum levels** | | ***VCAM1***  **mRNA expression** | | **MCP-1**  **serum levels** | | ***CCL2***  **mRNA expression** | | **ADMA**  **serum levels** | | ***PRMT1***  **mRNA expression** | |  |  |  |
| *Variable* | *r* | *p* | *r* | *p* | *r* | *p* | *r* | *p* | *r* | *p* | *r* | *p* |  |  |  |
| Duration of RA (years) | 0.157 | 0.59 | -0.291 | 0.34 | **0.542** | **0.04** | 0.446 | 0.15 | 0.325 | 0.22 | **0.645** | **0.01** |  |  |  |
| CRP (mg/dL) | 0.198 | 0.52 | -0.022 | 0.95 | 0.233 | 0.42 | 0.164 | 0.63 | **0.585** | **0.02** | 0.406 | 0.17 |  |  |  |
| ESR (mm/1^st^ hour) | 0.132 | 0.67 | 0.119 | 0.71 | 0.237 | 0.42 | -0.119 | 0.73 | 0.190 | 0.50 | 0.043 | 0.89 |  |  |  |
| DAS28-CRP | 0.423 | 0.13 | 0.108 | 0.72 | 0.270 | 0.33 | 0.030 | 0.92 | 0.443 | 0.09 | 0.229 | 0.43 |  |  |  |
| DAS28-ESR | 0.349 | 0.22 | 0.119 | 0.70 | 0.228 | 0.41 | -0.055 | 0.87 | 0.389 | 0.14 | 0.244 | 0.40 |  |  |  |
|  |  |  |  | |  |  |  | |  |  |  | |  |  |  |
| *Category* | *Mean ± SD*  *(ng/mL)* | *p* | *Mean ± SD* | *p* | *Mean ± SD*  *(pg/mL)* | *p* | *Mean ± SD* | *P* | *Mean ± SD*  *(µmol/L)* | *p* | *Mean ± SD* | *p* |  |  |  |
| RF^-^ | 1628.467 ± 1131.011 | 0.15 | 0.00015 ± 0.00011 | 0.42 | 583.600 ± 360.171 | 0.47 | 0.00085 ± 0.00048 | 0.65 | 0.489 ± 0.028 | 0.27 | 0.02729 ± 0.00948 | 0.15 |  |  |  |
| RF^+^ | 4024.387 ± 3132.251 |  | 0.00019 ± 0.00021 |  | 672.500 ± 257.898 |  | 0.00157 ± 0.00106 |  | 0.550 ± 0.080 |  | 0.06490 ± 0.07529 |  |  |  |  |
| ACPA^-^ | 6256 .000 | 0.54 | 0.00048 | 0.09 | 831.000 | 0.88 | - | - | 0.544 | 0.84 | 0.04247 | 0.79 |  |  |  |
| ACPA^+^ | 3470 ± 3030.412 |  | 0.00015 ± 0.00019 |  | 648.878 ± 270.692 |  | 0.00148 ± 0.00102 |  | 0.541 ± 0.080 |  | 0.05959 ± 0.07185 |  |  |  |  |
| VCAM-1: vascular cell adhesion molecule 1; MCP-1: monocyte chemoattractant protein-1; ADMA: asymmetric dimethylarginine; RA: rheumatoid arthritis; ILD: interstitial lung disease; CRP: C-reactive protein; ESR: erythrocyte sedimentation rate; DAS: disease activity score; RF: rheumatoid factor; ACPA: anti-cyclic citrullinated peptide antibodies. Significant results are highlighted in bold. | | | | | | | | | | | | |  |  |  |
