# Supplementary material for: Elevated VCAM-1, MCP-1 and ADMA serum levels related to pulmonary fibrosis of interstitial lung disease associated with rheumatoid arthritis
Source: Front Mol Biosci. 2022 Dec 19;9:1056121. doi: 10.3389/fmolb.2022.1056121 (PMC9806218; doi:10.3389/fmolb.2022.1056121)
Supplement: Supplementary file 4 [file Table4.DOCX]

| Supplementary Table S4. Relationship of VCAM-1, MCP-1 and ADMA serum levels as well as *VCAM1*, *CCL2* and *PRMT1* mRNA expression with clinical characteristics intrinsic of the rheumatic disease in RA-ILD^-^ patients. | | | | | | | | | | | | |  |  |
| --- | --- | --- | --- | --- | --- | --- | --- | --- | --- | --- | --- | --- | --- | --- |
|  | **VCAM-1**  **serum levels** | | ***VCAM1***  **mRNA expression** | | **MCP-1**  **serum levels** | | ***CCL2***  **mRNA expression** | | **ADMA**  **serum levels** | | ***PRMT1***  **mRNA expression** | |  |  |
| *Variable* | *r* | *p* | *r* | *p* | *r* | *p* | *r* | *p* | *r* | *p* | *r* | *p* |  |  |
| Duration of RA (years) | -0.169 | 0.47 | 0.431 | 0.10 | 0.041 | 0.86 | -0.055 | 0.83 | -0.318 | 0.15 | 0.328 | 0.15 |  |  |
| CRP (mg/dL) | 0.332 | 0.14 | 0.247 | 0.36 | 0.082 | 0.73 | -0.095 | 0.72 | 0.091 | 0.69 | 0.145 | 0.53 |  |  |
| ESR (mm/1^st^ hour) | 0.167 | 0.47 | 0.015 | 0.96 | -0.097 | 0.68 | 0.275 | 0.30 | -0.0007 | 1.00 | -0.026 | 0.91 |  |  |
| DAS28-CRP | 0.024 | 0.92 | 0.245 | 0.36 | 0.371 | 0.11 | 0.103 | 0.69 | 0.073 | 0.75 | 0.145 | 0.53 |  |  |
| DAS28-ESR | -0.015 | 0.95 | 0.155 | 0.57 | 0.189 | 0.42 | 0.297 | 0.25 | 0.031 | 0.89 | 0.131 | 0.57 |  |  |
|  |  |  |  | |  |  |  | |  |  |  | |  |  |
| *Category* | *Mean ± SD*  *(ng/mL)* | *p* | *Mean ± SD* | *p* | *Mean ± SD*  *(pg/mL)* | *p* | *Mean ± SD* | *P* | *Mean ± SD*  *(µmol/L)* | *p* | *Mean ± SD* | *p* |  |  |
| RF^-^ | 1284.093 ± 959.549 | 0.11 | 0.00346 ± 0.00026 | 0.31 | 390.808 ± 142.871 | 0.37 | 0.00075 ± 0.00028 | 0.34 | 0.497 ± 0.059 | 0.22 | 0.120 ± 0.097 | 0.91 |  |  |
| RF^+^ | 884.690 ± 388.974 |  | 0.00021 ± 0.00018 |  | 427.780 ± 108.866 |  | 0.00066 ± 0.00025 |  | 0.470 ± 0.061 |  | 0.116 ± 0.082 |  |  |  |
| ACPA^-^ | 966.990 ± 559.209 | 0.81 | 0.00035 ± 0.00029 | 0.19 | 434.044 ± 140.013 | 0.41 | 0.00074 ± 0.00036 | 0.87 | 0.498 ± 0.063 | 0.18 | 0.142 ± 0.101 | 0.34 |  |  |
| ACPA^+^ | 1225.307 ± 922.293 |  | 0.00026 ± 0.00019 |  | 389.421 ± 121.402 |  | 0.00069 ± 0.00023 |  | 0.477 ± 0.059 |  | 0.104 ± 0.081 |  |  |  |
| VCAM-1: vascular cell adhesion molecule 1; MCP-1: monocyte chemoattractant protein-1; ADMA: asymmetric dimethylarginine; RA: rheumatoid arthritis; ILD: interstitial lung disease; CRP: C-reactive protein; ESR: erythrocyte sedimentation rate; DAS: disease activity score; RF: rheumatoid factor; ACPA: anti-cyclic citrullinated peptide antibodies. | | | | | | | | | | | | |  |  |

***Supplementary Material***
